# Diagnosis, medication, and surgical management for patients with trigeminal neuralgia: a qualitative study

**DOI:** 10.1007/s00701-015-2515-4

**Published:** 2015-09-02

**Authors:** Matthew J. Allsop, Maureen Twiddy, Hilary Grant, Carolyn Czoski-Murray, Mark Mon-Williams, Faisal Mushtaq, Nick Phillips, Joanna M. Zakrzewska, Sue Pavitt

**Affiliations:** Leeds Institute of Health Sciences, University of Leeds, 101 Clarendon Road, Leeds, LS2 9LJ UK; Institute of Psychological Sciences, University of Leeds, Leeds, LS2 9JT UK; Department of Neurosurgery, Leeds General Infirmary, Great George Street, Leeds, LS1 3EX UK; Eastman Dental Hospital, UCLH NHS Foundation Trust, 256 Gray’s Inn Road, London, WC1X 8LD UK; School of Dentistry, University of Leeds, Worsley Building, Leeds, LS2 9JT UK

**Keywords:** Trigeminal neuralgia, Medication, Surgery, Management, Qualitative research

## Abstract

**Background:**

Trigeminal neuralgia (TN) is a serious health problem, causing brief, recurrent episodes of stabbing or burning facial pain, which patients describe as feeling like an electric shock. The consequences of living with the condition are severe. There is currently no cure for TN and management of the condition can be complex, often delayed by misdiagnosis. Patients’ qualitative experiential accounts of TN have not been reported in the literature. Capturing subjective experiences can be used to inform the impact of the condition on quality of life and may contribute to a better understanding of current clinical practice with the aim of improving patient care.

**Methods:**

Participants with TN (*n* = 16; 11 female), including those who have and have not undergone surgical intervention(s), took part in one of four focus groups. We conducted a thematic analysis within an essentialist framework using transcripts.

**Results:**

The impact of TN and treatment on the lives of participants emerged as four predominant themes: (1) diagnosis and support with TN, (2) living in fear of TN pain, (3) isolation and social withdrawal, and (4) medication burden and looking for a cure. Each theme is discussed and illustrated with extracts from the transcripts.

**Conclusions:**

Key issues to address in the management of patients with TN include continued delays in diagnosis, persistent side effects from medication, and a lack of psychological support. Developing strategies to enhance the management of patients with TN, informed by a biopsychosocial approach and multidisciplinary team working, is essential to enhancing the provision of current care.

## Introduction

Trigeminal neuralgia (TN) is a serious health problem, causing brief, recurrent episodes of stabbing or sharp facial pain, which patients describe as feeling like an electric shock [10]. Attacks can be triggered by any kind of movement or touch; they can occur at numerous intervals or continuously throughout the day [27]. Trigeminal neuralgia is rare, and hence it is difficult to obtain high-quality epidemiological data. Data from GP practices based in the United Kingdom (UK) outlined an incidence of 8 per 10,000 people per year and a lifetime prevalence of 0.7 per 100,000 people per year [15]. The consequences of living with the condition are severe. Often patients live in fear of pain, with daily functioning disrupted and quality of life impaired [26], typically presenting with higher levels of anxiety and depression [16].

There is currently no cure for TN and management of the condition can be complex, often delayed by misdiagnosis. The lack of any objective testing for TN remains a significant clinical problem, impairing clinical research [11]. Patients with orofacial pain may present to their dental practitioner where the origin is unclear, with pain subsequently treated as relating to teeth or surrounding tissue [12]. Once diagnosed, medication is often the first-line treatment for TN patients. Carbamazepine is recognized as the drug of choice [18, 27]. Pain severity compels many patients to use much higher doses, leading to significant cognitive and motor function side effects [14, 25]. A number of surgical management options are available for TN, but are typically only offered when medications fail or side effects are too severe [21]. One procedure, microvascular decompression, aims to preserve trigeminal nerve function, with all other procedures deemed destructive or ablative [27]. The effectiveness, mortality rates, and morbidities of these procedures are summarized in Table 1 (based on national guidelines) [6, 9, 19].

**Table 1:**
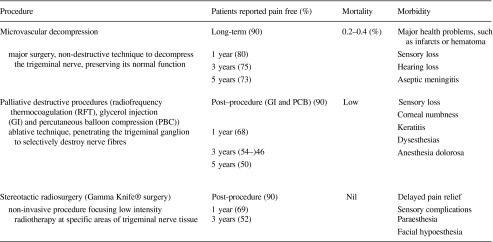
Surgical treatments for trigeminal neuralgia

Patients’ qualitative experiential accounts of TN have not been reported in the literature, but they could be used to report the impact of the condition on quality of life and may contribute to a better understanding of current clinical practice with the aim of improving patient care. While existing accounts of the patient’s journey through TN exist [26, 29], this is the first reported qualitative study into the experience of TN and the impact of treatment by patients diagnosed with TN.

## Methods

### Participants

Participants with TN (*n* = 16; 11 female), including those who have and have not undergone surgical intervention(s), took part in the study. Variation in the use of medication(s) to manage symptoms was identified across participants (see Table 2). The duration of time since initial experience of TN symptoms was very short for three participants, but more experienced a long delay in diagnosis with some lasting up to 10 years. Despite most participants (*n* = 13) having received surgery, a number (*n* = 5) were also continuing to use medication.

**Table 2:** Overview of participant demographics and treatment history

	Demographics				Treatment history								
					Current drug management						Previous surgical procedures		
Focus group	Participant	Sex	Age	Year of diagnosis	C	G	T	P	L	Other	GK	MD	PM
1	1	M	68	2009	**+**						X		X
	2	F	65	1998		O			O				X
2	3	F	**+**	1996	O						**+**		
	4	F	79	1998									XX
	5	F	45	2004			O			Pa; I		X	
	6	F	77	2007	O			O					XX
3	7	F	70	1997	O			O					XX
	8	M	66	2011							X	X	
	9*	M	60	2011	**+**						**+**		
	10	F	70	1999	**+**								X
	11*	F	79	1988	O	O					X		XXXXXX
4	12	F	54	2011									X
	13	F	70	2011							X		X
	14	M	67	2010							X		X
	15	F	72	2011									XX
	16	M	74	2007							**+**		

Participant:

* = participant has multiple sclerosis

+ = participants did not want to provide this information

O = participant reported current medication at time of focus group

X = each ‘X’ represents completion of procedure. Multiple use indicates number of times procedure was completed

Medical management:

*C* carbamazepine, *G* gabapentin, *T* tramadol, *P* pregabalin, *L* lamotrigine, *Pa* paracetamol, *I* ibuprofen

Surgical management:

Gamma knife (GM) = stereotactic radiosurgery using the gamma knife; microvascular decompression (MD) = microvascular decompression involving a craniectomy; percutaneous methods (PM) = radiofrequency thermocoagulation, glycerol rhizolysis, balloon compression, or local avulsion and cryotherapy of the peripheral branches

### Design

Capturing subjective experiences of the pain sufferer using a qualitative approach can make important contributions to evaluating and improving practice [17]. Four focus groups comprising 2–5 participants were completed, each lasting between 1 h 20 min and 2 h. The discussions were facilitated by two experienced qualitative health researchers with expertise in psychology (MA, MT). Focus groups were utilized because they do not assume pre-existing opinions on a particular topic, yet enable elicitations of opinions, ideas, and beliefs in a group setting. The researchers were therefore able to gain a better insight into both the common and diverse range of experiences of those living with TN.

### Participants and recruitment

Participants were identified from one neurosurgery department database. The clinical team identified participants aged >17 years, with a diagnosis of TN and capacity to consent themselves into the study from a list of outpatients from 12 months prior to the start of the study. A maximum variance sampling approach was used, intended to capture heterogeneity across participants. We sampled patients to ensure that we captured those with experience of a longer-term impact of TN (at least 2 years post-intervention) and those who have experienced recent surgery (in the last 6 months) or had surgery planned. All eligible participants were sent an information pack via post, which explained the purpose of the study, and contained an invitation letter.

A total of 40 invitations were issued. Those interested in participating could contact the research team via phone or using the response form and prepaid envelope included in the pack. A member of the research team contacted interested parties to arrange a suitable date for a focus group. The focus groups used a topic guide informed by research literature on the side effects of TN medication and experience of the clinical team. The topic guide asked about participants’ experience of TN, medication used, and any side effects (e.g., cognitive, behavioral), alongside the impact on daily living (e.g., family life, social life, employment). Focus groups were audio recorded and took place at the University of Leeds. Ethical approval was provided by the National Research Ethics Service Committee for Yorkshire & the Humber–Leeds Central, research ethics committee (reference number 12/YH/0394).

### Data analysis

We conducted a thematic analysis within an essentialist framework [5], which rests on the assumption that participants described personal experiences, perceptions and understandings of living and coping with TN and treatment provision. Each focus group was transcribed verbatim. Participants were offered the opportunity to review the transcripts but none took up this offer. Transcripts were read several times and the audio recording of each transcript listened to simultaneously. The transcripts were summarized in written notes to assist a thorough understanding of each participant’s experiences. Categories were identified and condensed in order to retain original expressions, and each category given a label. Three researchers were involved in data analysis. One researcher coded all transcripts; the other two reviewed all transcripts and coded sections of the data to ensure consistency in coding. All three met and discussed the development of the thematic themes and write up. NVivo 9 software (QSR International Pty Ltd, Australia) was used for carrying out further coding and facilitating thematic analyses. Common codes were created by comparing content across all four focus groups in order to create common codes relating to the impact of TN on quality of life. The end point of the analysis was to illustrate each theme by means of representative quotations from the data.

## Results

The impact of TN and treatment on the lives of participants emerged as four predominant themes [1]: diagnosis and support with TN [2], living in fear of TN pain [3], isolation and social withdrawal, and [4] medication burden and looking for a cure. Each theme will be discussed and illustrated with extracts from the transcripts collated in Table 3, with reference to relevant quotes indicated in the text.

**Table 3:** Quotations extracted from transcripts

Theme	Reference	Text, participant, and participant sex (three dots (…) indicate that text has been omitted)
Diagnosis and support with TN	1	I started about 10 years ago, the first 2 years they were diagnosing me with sinus problems. FG3, female
	2	I had all my fillings taken out and put back in and teeth taken out… I had Botox… no results at all until they suddenly decided that it was TN… FG2, female
	3	I could lie down and go to sleep now; it’s a real problem of fighting the tiredness all day long. FG3, male
	4	When you’re in that pain, you know, your life is just wiped out… FG3, female
	5	…once you start getting low like that, when the tears start, it’s very hard to come out of it because you’re on a spiral down… FG2, female
	6	It’s like....I always feel …I’ve got a demon sitting on my shoulder (laughter)… What have I done?…It’s as if somebody has just hit you in the face for no reason whatsoever and you just want to hit back, you know. FG4, female
	7	To me, it’s like someone had a knife and just kept going [stabbing] in to part of my face all the time. … I didn’t have minutes of the day when it wasn’t there. FG2, female
	8	… anybody with TN, when it gets to the stage where they[we] can’t talk, we need to see a [consultant] surgeon as quickly as possible… FG1, female
Living in fear of TN pain	9	I don’t clean my teeth a lot because I’m scared to death of setting it [the pain] off. FG3, male
	10	I couldn’t lie down, the covers touching [the face] set it off, so I didn’t sleep or I had to sleep in an upright position. FG2, female
	11	I’m in pain with it, but I have to keep going, otherwise I’m giving in to it… FG4, female
	12	I lost two stones because I couldn’t eat; I used to live on Maltesers and full cream milk…that was my diet. I could put Maltesers in my mouth and slowly let them dissolve… FG1, male
	13	…a couple of whiskies …that seemed to make the medication work better… FG3, female
	14	I couldn’t even have my tea in front of my children or my husband because I felt I looked like a freak. …you’re in pain and feel like you’re contorted. FG2, female
Isolation and social withdrawal	15	I just got a personality change and was very sort of serious and quiet, I can’t explain it, all my friends noticed… I was like a zombie. FG3, female
	16	I wouldn’t do social things, in case it [the pain] started while I was out… FG2, female
	17	If I couldn’t get the pills I would commit suicide. FG3, male
	18	I would probably have put a bullet through my brains because the pain was so intense. FG2, female
	19	Imagine, it’s your daughter’s … birthday party, you can’t speak, and you can’t eat, every time you try to speak [it’s like] someone smacks you straight in the mouth… so you’re not going to go are you? FG1, male
	20	I used to pick up my grandchildren and give them a bear hug, but because the pain gradually got worse over the years, I had to stop doing it, and that definitely put a distance between us. FG3, female
Medication burden and looking for a cure	21	I was relying on those drugs because I believed it was taking some of the pain away and was scared to come off them, in case it was, but still I was in severe pain… too many drugs and still in pain. FG2, female
	22	I’m taking amitriptyline and pregabalin and I had gabapentin before that and co-codomol but they don’t do anything, it’s [pain] just there all the time… FG3, female
	23	Well I actually overdose on gabapentin and I take 1200 mg three times a day plus… when you’re in that pain you’ll take anything. FG3, male
	24	I’ve done maximum, you know, …couldn’t bear it anymore, and I have actually I must admit, I have…carbamazepine, actually, and pain killers … I had it so bad one night… FG4, female
	25	[the doctor] said to me: “You’re toxic you’ve got far too much carbamazepine in you… I’d also started with eczema, which the dermatologist thought was …a reaction to the carbamazepine. FG2, female
	26	Yes, that [Tegretol retard] did inflame my liver; I was really poor with it, so I came off of it quite quickly and then, of course, I was in agony because I was in-between pills… FG3, female
	27	When I go onto the drugs it normally gets rid of the pain … [but] the drugs exacerbate all my MS [multiple sclerosis] symptoms… so, If I’m on a preventive dose I don’t go out of the house, if I’m on the medication, I’m stuck there. FG4, female
	28	… I stopped taking them [the tablets] like that, I suffered for it, but… I thought: “I’m sick of taking tablets…” Taking that many tablets you forget whether you’ve taken them or not. So I came straight off them… FG1, male
	29	I gave up doing gas work, I couldn’t guarantee that I could concentrate on it enough to do it… I chopped [off] the ends of three fingers, I wasn’t concentrating … I was thinking about the pain instead of what I was doing… FG3, male
	30	I had people accusing me of being a “druggie”…work colleagues saying: “there’s nothing wrong with you, you’re just a druggie”, you’re on drugs… FG2, female
	31	I just couldn’t keep up, my reactions had slowed down… it would feel as if I were being pushed out, so I just gave my notice. FG1, female
	32	I did run into the back of three cars while driving… I shouldn’t have been driving but I was reluctant to give up work and work involved travel. FG2, female
	33	I couldn’t calculate, I lost my mental arithmetic, it disappeared I just could not think…So yeah it did affect me…FG3, female
	34	Simple everyday things like that I should have known had just gone and it didn’t matter how I tried I just couldn’t bring them back. FG2, female
	35	I have a feeling of swaying all the time, as if I was going to sort of fall over in slow motion… FG3, female
	36	I’m sort of not connecting between my brain and what I’m doing, and sometimes I can write and think…no problem, other times I can’t… FG1, female
	37	…surgery, it was never mentioned for me when it [the pain] was bad…when I was kind of drug-controlled. FG2, female
	38	I just wanted to cut down on the medication… it [surgery] did that… It got rid of the pain… it’s just uncomfortable now. I can live with uncomfortable. FG2, female
	39	…once I had this done, it was wonderful, all the pain had gone I was back to normal, it’s really really nice… when you can’t, talk, eat, socialize, it takes a lot way from you.. FG1, male

### Diagnosis and support with TN

This theme describes the diagnosis and support provided for TN. Most participants reported a delay in an accurate diagnosis alongside difficulties in adjusting to the diagnosis of TN. Participants described the severity of TN pain and their needs for health professional support during symptomatic episodes.

Misdiagnosis or extended exploratory investigations (e.g., dental work) occurred for most participants prior to receiving an accurate diagnosis. This was viewed as a significant barrier to accessing appropriate care resulting in many ineffective interventions. The theme also describes the impact of severe pain experienced with the condition (ref 1 and 2). Participants often accessed standard treatments but they reported that they still experienced pain, which they found imposed significant limitations on their daily lives. As well as constantly feeling fatigued, participants also expressed a sense of loss (ref 3 and 4).

These descriptions illustrate the distress that is a common consequence of TN. One reason for this is that participants become overwhelmed by the severity of the pain, and associated difficulties, and so withdraw, socially. For one participant, it is difficult to halt feelings of pessimism at those times (ref 5). Another participant indicated feelings of anger and resentment at having TN. She momentarily implicates herself, as if pondering whether she is in some way responsible for her illness (ref 6).

The analogy of being suddenly struck in the face or the mouth was used by many participants to describe the onset of episodes of debilitating pain that last for varying lengths of time and recur in phases, before easing (ref 7). Because of the sudden and severe nature of TN pain, participants reported a need for urgent referral to their consultant, when they became symptomatic (ref 8). This would be the preferred pathway, rather than routine appointments, when asymptomatic. Participants’ accounts illustrate the considerable burden the sudden nature of TN pain imposes on participants. Between attacks, participants were fearful that the pain could return at any time, positioning them as suffering from pain and the fear of pain.

### Living in fear of TN pain

This theme captures the individual impact of TN on the lives of participants and the disruption it causes as they try to negotiate daily life. Addressing aspects of quality of life, sub-themes relating to personal care, diet, impact on emotions, relationships with family and friends, were discussed in all four focus groups.

Participants reported a range of avoidance behaviors, affecting the face, since the onset of TN. Fear of pain episodes prevented participants from engaging in aspects of personal care, which they had experienced as pain stimuli (ref 9 and 10). Other participants, however, expressed the attitude that they would continue trying to lead their lives as normal, even with the potentially disruptive nature of their illness (ref 11). Despite being determined to continue as normal, participants were forced to make adjustments in important areas of their lives. Eating presents a major problem in TN and participants’ reports highlighted a dilemma which placed them between needing to avoid chewing—in order to prevent pain—and patterns of disordered food and drink consumption, which put participants at risk of nutrient deficiency. While one participant relied on liquidized food, another adopted an extremely restricted diet (ref 12). Other participants reported including alcohol in their diet, to self-medicate and to help them cope with the pain (ref 13). Participants’ difficulty eating, while in pain, was revealed as anxiety-provoking and as having implications for wider social interaction (ref 14).

### Isolation and social withdrawal

Emotional difficulties, manifested in feelings of isolation and withdrawal from social interaction, were reported by participants in all four groups. Often social isolation arose from decisions by participants to withdraw as a direct consequence of pain, or fear of pain. The descriptions of two participants illustrated experiences of social anxiety (ref 15 and 16).

Depressive symptoms were also indicated by two participants, the second, awaiting surgery, who made explicit the extent of their emotional distress (ref 17 and 18). Families and friends have an important effect on the well-being of participants as they manage their illness. Most participants reported that relatives were supportive, but pain severity induced isolation and made social interaction difficult, even with family (ref 19 and 20). Avoidance of certain aspects of daily living and of social situations is presented as central to the impact of TN. Despite the strength of relationships within families, the pain and unpredictability of the condition cause difficulties in adjustment, for all parties.

### Medication burden and looking for a cure

Participants in all four focus groups reported limitations in pharmacological management. They identified a range of issues that characterized treatment failure, and as can be seen by Table 2, over time, most had experienced several changes in medication. The majority expressed a sense of being overwhelmed by the side effects of drug treatment, in addition to continuing pain (ref 21 and 22). Many participants directed the problem of pain towards attempts to self-manage medication, continually assessing their needs and fluctuating between dosages in attempts to establish an effective regimen and exert a degree of control over their pain. Two participants revealed that they had intentionally over-medicated because of pain severity (ref 23 and 24).

For most participants, the attempt to self-manage medication involved a constant struggle to balance their need for optimal pain relief against their wish to avoid dose-related side effects. All participants reported extreme tiredness, with some developing additional health issues (ref 25 and 26).

Even when drug intake produced anticipated benefits, participants still encountered challenges to their quality of life, with increased treatment side effects being accepted as trade-offs for achieving pain relief from TN (ref 27). Participants reported experiencing long-term medication use to be as burdensome as living with the chronic nature of TN itself. Many felt forced to make difficult choices about whether to endure side effects or risk the pain associated with TN (ref 28).

When taking chronic pain medication, participants encountered different impacts that were defined by their initial experiences of treatment. Participants often felt overwhelmed by the drugs they were prescribed, they worried they would not work, and experienced distressing side effects and associated health issues. Participants reported a number of medication-related cognitive impairments, which caused additional difficulties in everyday functioning. Employment was one of the key areas in which participants were affected by the debilitating combination of chronic pain and side effects of medication. One participant reported leaving his job after an accident caused him to question the safety of his work (ref 29). A participant reported being subjected to accusations of drug addiction when side effects of pain medication caused fatigue, along with speech and mobility problems (ref 30). Another participant reported that medication side effects affected her performance on accountancy computing tasks at work. The difficulties she encountered led her to leave her job (ref 31).

Difficulties caused by side effects were broad. Driving was an environment in which participants experienced concentration difficulties. One participant reported that she had been suffering side effects when she caused several minor driving collisions (ref 32). Mental arithmetic was more difficult for participants when they were taking medication (ref 33). Memory was another key area of difficulty. Three reported problems with recall of numbers and also recall of words and names. Additionally, skills such as following a knitting pattern (ref 34) were impaired. Two participants reported motor function difficulties, which affected their balance. The same participants experienced problems with fine motor control, with both describing how they were often unable to co-ordinate their actions for handwriting (ref 35 and 36).

As all focus group participants had been on medication for significant periods of time, they were all able to identify ways in which the high doses they used had affected their cognitive functioning. Some participants had undergone at least two surgical procedures, hoping to be cured. Resolution of TN is challenging but surgery can improve pain, and all participants reported less medication use, post-surgery. While not completely off medication, because of fears of pain recurrence, participants reported less tiredness and better functioning. Although participants had experienced negative effects whilst using medication, they had been compliant because surgical interventions had not been offered (ref 37). Having taken significant doses of medication for long periods of time, participants were also knowledgeable about medication side effects and have realistic expectations of surgery (ref 38).

Where surgical procedures had been performed, positive outcomes were reported. For example, participants who had undergone GK treatment reported benefits to their everyday lives. In contrast with their former feelings of isolation, participants valued the symptom relief achieved by this procedure as enabling them to re-engage with aspects of life that they considered important (ref 39).

## Discussion

These accounts contribute the first qualitative study into the impact of living with TN, outlining the impact of TN pain and subsequent medications on the social lives of participants. The experience described by participants is one of uncertainty and fear. In line with narratives provided around the experience of TN [26, 29] participants drew on experiences of sudden, crippling pain. There was also marked variation in lengths of time in which patients did not know the cause of their pain before receiving a diagnosis. Most participants presented to dentists, where knowledge gaps in chronic orofacial pain have been identified in both general dental practitioners and dental specialists [2]. This, teamed with the lack of any objective testing for TN may in part explain the seemingly random likelihood of receiving an accurate and timely diagnosis. For patients with multiple sclerosis, a shorter duration to diagnosis was discussed, perhaps aided by a 20-fold higher presence of TN in this patient group than in the general population and recognition of the condition by neurologists [20].

The study recruited participants through a large neurosurgery department in the north of England. The experiences of services by participants in this study are limited to the region covered by the department, albeit a large regional tertiary referral centre offering a full range of surgical management options. Participants were a subgroup of TN patients interacting with a neurosurgeon via an outpatient clinic and further exploration of patient management across a range of sites would contribute to a broader understanding of service provision for patients with TN. The accounts depicted in this study have arisen from analysis by two psychologists (MA and MT) with involvement of a multi-disciplinary team including a neurosurgeon and applied health researchers. We made ensured involvement of additional researchers in the focus group and analysis process (HG, FM) to enhance the rigor and transparency of analysis.

The narratives of participants serve to align the responses of TN patients with individuals who report chronic pain. The far-reaching effects and solitary experience of chronic pain [24] and impact of pain on the quality of social and working lives [23] were reported. Dietary and behavior changes as an approach to avoiding a wide variety of stimuli were also reported. Such avoidance both directly (e.g., physical activities) and indirectly (e.g., social activities) has been documented for those with chronic pain [3]. Participants’ reported approaches to managing pain are not abnormal, but aligned with people managing severe pain in their lives.

The use of medication for pain by participants with TN was often described as prophylactic, taken regularly and increased when pain occurred. The severity of the pain meant that patients would persist for long periods of time using medication, despite all drugs having the potential to result in neurological side effects such as drowsiness, ataxia, and diplopia at higher doses [27]. Pain guidelines typically emphasize self-management approaches, but provide little information on how to facilitate it [7]. Specialist services such as pain clinics have demonstrated that supporting medication use can improve self-management of pain medication [8]. While many participants in this study had close interaction with TN specialists, their lives continued to be affected by the condition with reports of underdosing and overdosing of prescribed pain medication as part of a process for seeking effective management of their pain. At one center the research team uses a clinical nurse specialist with prescribing rights to provide further advice about medication which has been evaluated very positively.

For TN, defining what good management is, and what can be expected from pharmacological and surgical management may support patient decision making from diagnosis. An attempt to look at how patients make decisions was hampered by lack of good quality evidence but suggested patients preferred surgical options [22]. While pharmacological management is the first line treatment for TN [27], many participants reported multi-pharmacy, alongside impaired cognitive and motor skills and subsequent consequences (work-related injuries and road accidents). Detection of these is difficult, as they are not picked up on routine medical reviews. For participants who had experienced pharmacological and surgical treatments, low mood, suicidal ideation, anger and self-blame, alongside diminished relationships with friends and family were reported. Psychological treatments are not a routine component in the treatment of TN, although they feature in the management of complex orofacial pain conditions, such as temporomandibular joint disorders, for which cognitive behavioral therapies have been shown to be effective [1, 13]. The research team has piloted a cognitive behavior program which includes a talk by a medical expert exclusively for TN patients to improve their coping strategies’ and it is currently being evaluated. Surgical interventions were offered to participants who had been experiencing pain that was refractory to pharmacological management, or where quality of life was significantly compromised. The place of surgical options later in the care pathway requires many patients to experience months and years of complex management of their pain, high levels of pain medication, and problematic side effects. Some centers do offer early neurosurgical opinion so patients are more aware of the options open to them and it enables the pathway to be accessed quicker in an emergency [28]. Alongside this, research is exploring new approaches to the management of severe cases such as the creation of specialist regional centers for chronic orofacial pain [4].

This study highlights the considerable impact on quality of life of both unpredictable episodic pain and complications from treatment which can occur after both pharmacological and surgical intervention. Developing strategies for addressing these key issues in the management of patients with TN are essential to enhance the provision of current care and require a more biopsychosocial approach using multidisciplinary teams.

## References

[1] Abrahamsen R, Baad-Hansen L, Zachariae R, Svensson P (2011). Effect of hypnosis on pain and blink reflexes in patients with painful temporomandibular disorders. Clin J Pain.

[2] Aggarwal VR, Joughin A, Zakrzewska JM, Crawford FJ, Tickle M (2011). Dentists’ and specialists’ knowledge of chronic orofacial pain: results from a continuing professional development survey. Prim Dent Care.

[3] Asmundson GJ, Norton PJ, Norton GR (1999). Beyond pain: the role of fear and avoidance in chronicity. Clin Psychol Rev.

[4] Beecroft EV, Durham J, Thomson P (2013). Retrospective examination of the healthcare ‘journey’ of chronic orofacial pain patients referred to oral and maxillofacial surgery. Br Dent J.

[5] Braun V, Clarke V (2006). Using thematic analysis in psychology. Qual Res Psychol.

[6] Cruccu G, Gronseth G, Alksne J, Argoff C, Brainin M, Burchiel K, Nurmikko T, Zakrzewska JM, American Academy of Neurology S, European Federation of Neurological S (2008). AAN-EFNS guidelines on trigeminal neuralgia management. Eur J Neurol.

[7] Dorflinger L, Kerns RD, Auerbach SM (2013). Providers’ roles in enhancing patients’ adherence to pain self-management. Transl Behav Med.

[8] Gatchel RJ, Okifuji A (2006). Evidence-based scientific data documenting the treatment and cost-effectiveness of comprehensive pain programs for chronic nonmalignant pain. J Pain.

[9] Gronseth G, Cruccu G, Alksne J, Argoff C, Brainin M, Burchiel K, Nurmikko T, Zakrzewska JM (2008). Practice parameter: the diagnostic evaluation and treatment of trigeminal neuralgia (an evidence-based review): report of the Quality Standards Subcommittee of the American Academy of Neurology and the European Federation of Neurological Societies. Neurology.

[10] Headache Classification Subcommittee of the International Headache Society (2013). The International Classification of Headache Disorders, 3rd edition (beta version). Cephalalgia.

[11] Ibrahim S (2014). Trigeminal neuralgia: diagnostic criteria, clinical aspects and treatment outcomes. A retrospective study. Gerodontology.

[12] Law AS, Lilly JP (1995). Trigeminal neuralgia mimicking odontogenic pain. A report of two cases. Oral Surg Oral Med Oral Pathol Oral Radiol Endod.

[13] Litt MD, Shafer DM, Kreutzer DL (2010). Brief cognitive-behavioral treatment for TMD pain: long-term outcomes and moderators of treatment. Pain.

[14] Loring DW, Marino S, Meador KJ (2007). Neuropsychological and behavioral effects of antiepilepsy drugs. Neuropsychol Rev.

[15] MacDonald BK, Cockerell OC, Sander JW, Shorvon SD (2000). The incidence and lifetime prevalence of neurological disorders in a prospective community-based study in the UK. Brain.

[16] Macianskyte D, Januzis G, Kubilius R, Adomaitiene V, Sciupokas A (2011). Associations between chronic pain and depressive symptoms in patients with trigeminal neuralgia. Medicina (Kaunas).

[17] Mitchell LA, MacDonald RA (2009). Qualitative research on pain. Curr Opin Support Palliat Care.

[18] National Institute for Health and Care Excellence (2013) NICE clinical guideline 173: Neuropathic pain—pharmacological management: the pharmacological management of neuropathic pain in adults in non-specialist settings https://www.nice.org.uk/guidance/cg173. Accessed 19 May 2015 25577930

[19] National Institute for Health and Clinical Excellence (2004) Stereotactic radiosurgery for trigeminal neuralgia using the Gamma Knife. NICE interventional procedure guidance 85. https://www.nice.org.uk/guidance/ipg85. Accessed 19 May 2015

[20] O’Connor AB, Schwid SR, Herrmann DN, Markman JD, Dworkin RH (2008). Pain associated with multiple sclerosis: systematic review and proposed classification. Pain.

[21] Obermann M (2010). Treatment options in trigeminal neuralgia. Ther Adv Neurol Disord.

[22] Spatz AL, Zakrzewska JM, Kay EJ (2007). Decision analysis of medical and surgical treatments for trigeminal neuralgia: how patient evaluations of benefits and risks affect the utility of treatment decisions. Pain.

[23] Tolle T, Dukes E, Sadosky A (2006). Patient burden of trigeminal neuralgia: results from a cross-sectional survey of health state impairment and treatment patterns in six European countries. Pain Pract.

[24] West C, Stewart L, Foster K, Usher K (2012). The meaning of resilience to persons living with chronic pain: an interpretive qualitative inquiry. J Clin Nurs.

[25] Zaccara G, Cincotta M, Borgheresi A, Balestrieri F (2004). Adverse motor effects induced by antiepileptic drugs. Epileptic Disord.

[26] Zakrzewska JM, Association TN (2006) Insights: facts and stories behind trigeminal neuralgia Trigeminal Neuralgia Association, Gainesville

[27] Zakrzewska JM, McMillan R (2011). Trigeminal neuralgia: the diagnosis and management of this excruciating and poorly understood facial pain. Postgrad Med J.

[28] Zakrzewska JM, Linskey ME (2014). Trigeminal neuralgia. BMJ.

[29] Zakrzewska JM, Padfield D (2014). The patient’s journey through trigeminal neuralgia. Pain: Clin Updates.

